# Chasing shadows: Investigating X chromosome mediation in late-onset Alzheimer’s disease

**DOI:** 10.36922/an.3122

**Published:** 2024-06-14

**Authors:** Carmel Armon, Lisa A. Cannon-Albright, Kristina Allen-Brady, Sharon Wolfson

**Affiliations:** 1 Department of Neurology, Loma Linda University School of Medicine, Loma Linda, California, United States of America; 2 Department of Neurology, Tel Aviv University School of Medicine and Shamir (Assaf Harofeh) Medical Center, Zerifin, Israel; 3 Genetic Epidemiology, Department of Internal Medicine, University of Utah School of Medicine, Salt Lake City, Utah, United States of America

**Keywords:** Utah Population Database, Late-onset Alzheimer’s disease, Maternal inheritance, Paternal inheritance, X chromosome, Risk factors

## Abstract

Alzheimer’s disease (AD) is a major cause of dementia. While maternal inheritance has been recognized for late-onset AD (LOAD), risk factors have not been identified consistently on the X chromosome. We recently developed a new method to identify an apparent risk of 70% mediated by the X chromosome in newly-presenting cognitive disorders clinic patients with amnestic mild cognitive impairment (aMCI) or early LOAD with unilateral parental lineage for AD or dementia. We sought to confirm our preliminary findings in the Utah Population Database (UPDB). We obtained previously published aggregate data on the risk of AD in the UPDB based on family history, stratified the data by the sex of the proband, and analyzed them using the new method. The X chromosome-attributable relative risk was estimated by calculating the following: Odds ratio (OR) = (women with paternal lineage: Women with maternal lineage)/(men with paternal lineage: Men with maternal lineage). The proportion of genetic risk attributable to the X chromosome is equal to (OR-1)/OR. The analysis did not reveal any risk mediated by the X chromosome, and the null result could be attributed to methodological limitations. Factors that impact the initial or early presentation (incidence) of LOAD, which are appropriate for consideration as risk factors, may not be detectable in a (prevalent) population of deceased individuals. Thus, epidemiological evidence for the contribution of the X chromosome to the development of LOAD will need to be sought prospectively in incident patient populations with newly diagnosed, biologically-confirmed aMCI or LOAD.

## 1. Introduction

Alzheimer’s disease (AD) is a major cause of dementia worldwide and imposes a great financial and social burden. The incidence of AD is projected to rise, particularly in developed countries, with the aging of the population due to the loss of competing causes of mortality.^1^ AD is characterized pathologically by extracellular accumulation of amyloid-β (Aβ) peptides generated by the cleavage of amyloid pre-cursor protein, strings of hyperphosphorylated tau proteins accumulating inside neurons known as neurofibrillary tangles, and neuronal loss.^2^ However, the underlying molecular mechanisms leading to the disease are poorly understood, and a study of brain samples from AD patients reveals differential changes in gene expression in neuronal and glial populations, with astrocytes and microglia displaying the greatest transcriptomic impacts.^3^ Three genes (*APP*, *PRES1*, *and PRES2*) have been found to account for the autosomal dominant inheritance of an early-onset form of the disease. The pathogenesis of late-onset AD (LOAD) also has a genetic basis. Prominent among the risk factors for LOAD are having one or more copies of the e4 allele of the *APOE* gene and a family history for the disease. By comparison, the risk conferred by more than 40 other genes/loci linked to AD^4^ is minor. Variants in exon 2 of *TREM2*, which encodes the triggering receptor expressed on myeloid cells 2 protein, are more frequent in patients with AD than in controls. Of these, the most commonly associated variant, rs75932628 (encoding R47H), shows a highly significant association with AD.^5^

A study based on the Utah Population Database (UPDB), which contains death certificate data,^6^ has contributed additional information concerning disease status in second-degree relatives (SDR) and third-degree relatives (TDRs), which is helpful for assessing the relative risk (RR) of AD occurrence in probands, beyond what could be determined by considering first-degree relatives (FDRs) alone. The study showed divergent directions for the effect of parental lineage on the risk of AD in probands when considering the degree of affected relatives.^6^ The estimated RR for an individual with an affected father exceeded the 95% confidence interval (CI) for the estimated RR for individuals with an affected mother. Individuals with an affected maternal SDR (mother’s brother or sister) had a significantly elevated RR, which was not observed in individuals with an affected paternal SDR (father’s brother or sister). RR for probands with TDRs (first cousins) affected was comparably elevated for maternal or paternal family history.

When analyzing the RR based on the sex of the probands, given an equivalent family history, men were at higher risk for AD than women.^6^ Some of these observations appear discordant from other published studies. Maternal inheritance has long been identified in LOAD,^7,8^ with the risk manifesting not only in symptomatic individuals^7^ but also in cognitively normal offspring, who were evaluated through imaging^8^ or screening for AD biomarkers.^9^ In contrast, based on general (mostly sporadic) population-based, age-specific data, the observation of greater risk for AD in men when there is an affected relative (as determined by death certificate data linked to genealogy) is concordant with the observed increased incidence in men of amnestic mild cognitive impairment (aMCI) – an early manifestation of AD.^10,11^ In women, the greater life-time risk and prevalence of AD observed is accounted for by their longer lifespan.

Despite the evidence that the sex of patients and their positive family history affect the estimated risk of developing sporadic and familial LOAD, no risk loci had been found consistently on the X chromosome until recently. Epigenetic mechanisms offer an explanation of how the X chromosome might be involved in the pathogenesis of AD.^12^ A recent publication^13^ has identified an X-linked ubiquitin-specific peptidase 11 that increases tauopathy vulnerability in women. An additional locus that provides sex-specific resilience to AD is located on chromosome 10.^14^ Differential effects of X chromosome gene expression on cognition and AD pathology have also been described.^15^ In particular, “X chromosome gene expression assessed by RNA sequencing was associated with cognitive change in women but not men in a manner independent of AD pathology. In contrast with cognition, X chromosome gene expression was associated with neuropathologic tau burden in men but not women”^15^ (p.1250). A second X chromosome has been shown to confer resilience against disease emergence in mouse models of AD, possibly mediated by the candidate gene *Kdm6a* located on the X chromosome.^16^ The role of the X chromosome in mediating the risk of AD remains enigmatic and may be mediated by loci on the chromosome itself or other chromosomes, or may depend on the degree of gene expression.

A new method has been proposed to address the impact of the X chromosome on developing AD which is independent of the precise pathway by which the risk might be conferred. It focused specifically on the sub-population with LOAD and unilateral parental lineage history for the disease.^17^ The novelty of the method is that it uses the sex of the affected proband and the parental lineage of the affected relatives to identify the chromosomes that might be involved in transmitting the risk for developing AD to the proband, then isolates the risk that may be mediated by the X chromosome. Specifically, an affected female proband can inherit the genetic contribution to her risk of developing AD via the 22 autosomal chromosomes and/or the X chromosome, regardless of whether the affected relatives are on the father’s or the mother’s side because she inherits 22 autosomal chromosomes and an X chromosome from each parent. In contrast, an affected male proband can inherit the genetic contribution to the risk of developing AD via the 22 autosomal chromosomes and/or the X chromosome if the risk arises from the mother’s side, but the genetic contribution to the risk can be transmitted only via the 22 autosomal chromosomes if the risk arises from the father’s side. This approach assumes that the risk for AD mediated by the genes on the Y chromosome is negligible. This may be an oversimplification. By counting the numbers of affected probands with unilateral parental lineage in a population-based sample, stratified based on proband sex and lineage sex, it is possible to calculate an odds ratio (OR) to isolate the contribution to risk mediated by the X chromosome.^17^ This method does not assume that all the risk in these patients is genetically-mediated. The details, assumptions, and limitations of this approach are elaborated in the Methods section. When applied to a small, single-center Cognitive Disorders Clinic sample of convenience, mainly consisting of incident patients, the estimated proportion of genetic risk borne by the X chromosome was as high as 70%, with a broad CI.^17^ The limitations of these preliminary findings have been recognized,^17^ and it is necessary to replicate and validate these findings.

In this study, we sought an available dataset of patients with AD who had a family history documented adequately to permit application of the new method^17^ and replication of the original analysis. We attempted to access one large database, but the information needed was not available, as family history was documented only as positive or negative without further elaboration regarding parental lineage. We then explored the possibility of utilizing the UPDS. The UPDB is a population-based dataset of uniformly defined patients and family histories and has already analyzed its data to demonstrate the impact not only of FDRs but also of SDRs and TDRs on the risk of AD.^6^ Proband sex-specific data were available for Table 1 of the original study.^6^ We analyzed the sex-specific data using the new method^17^ to replicate, if possible, the original observations.^17^

## 2. Methods

### 2.1. Overview

This section has two major components. First, we describe the UPDB, and how cases were selected for the present analysis. The UPDB, case selection, and RR estimation have been described previously in detail^6^ and are summarized here, focusing on the data used for the present analysis. Next, we describe the new method of analysis that we applied to the UPDB data.

#### 2.1.1. UPDB data

The UPDB encompasses the computerized genealogy of the Utah pioneer founders from the 1800s, and their descendants to the modern day, linked to many demographic and health-related data registries.^18^ Of the 11 million Utah-connected individuals represented in the UPDB, approximately 1.3 million have genealogy data for at least 12 of their 14 immediate ancestors spanning at least three generations. The genealogy data in the UPDB analyzed for this study have been linked to computerized Utah death certificates issued from 1904 to 2014. Cause of death was coded using the International Classification of Diseases (ICD) revisions 6 – 10, depending on the year of death.^6^ Only individuals with genealogy-linked death certificates coded with ICD-9 or ICD-10 were included because AD first appeared as a coded cause of death in ICD-9.

#### 2.1.2. Case selection

Individuals with an ICD code for AD as a primary or contributing cause of death (ICD-9: 331.0; ICD-10: F00 or G30) were identified as AD cases. This definition was also used previously.^19^ All individuals with ancestral genealogy and a linked Utah death certificate were analyzed to estimate population rates for AD in the UPDB (n = 270,818). Cohort-specific rates for AD were estimated for cohorts classified by sex, 5-year birth year range, and birthplace (Utah or not) by determining the ratio of the number of AD deaths in a cohort to the number of Utah deaths in the cohort.^6^

#### 2.1.3. Estimation of RRs

In the original report,^6^ RRs were estimated for many different family history constellations, based on the number of affected FDRs, SDRs, and TDRs, age at death from AD, sex of proband, and maternal vs. paternal inheritance. Probands in the UPDB might be either affected (with AD) or unaffected. FDRs include parents, offspring, and siblings; SDRs are the FDRs of FDRs; and TDRs are the FDRs of SDRs. For each constellation, all probands with the specific family history constellation in the UPDB were identified. The estimated RR for each specific family history constellation was calculated as the ratio of the observed number of AD cases among the probands to the expected number of AD cases among the probands; 95% CIs for the RR were calculated using the method proposed by Agresti.^20^ The expected number of AD cases among the probands was estimated by counting all the probands by cohort, multiplying the number of probands in each cohort by the cohort-specific rate of AD, and summing over all cohorts.^6^

#### 2.1.4. Specific summary data used in the present report

The present analysis employed data presented in Table 5 of the original report,^6^ which were made available separately for female and male probands. The same proband may be considered several times, for example, with mother’s brother, father’s brother, mother’s sister, or father’s sister. The present analysis relied on the proband numbers provided.

### 2.2. Statistical analysis

The data in this study were analyzed in accordance with the previously described methods.^17^ In the original report,^17^ all the affected individuals had symptomatic disease: aMCI or early AD. In contrast, the subjects included in this analysis are individuals documented in the UPDB who were AD-verified per their death certificates.

We used the affected individual’s sex chromosomes (XX or XY) and the parent’s sex chromosomes (XX or XY) to infer which chromosomes may be involved in transmitting the risk of AD: 22A+X (from mothers to all offspring and from fathers to daughters) or 22A+Y (from fathers to sons). For this calculation, we assumed the risk borne by the Y chromosome to be negligible and removed it from the calculation (it is not reflected in the table). The X chromosome-attributable RR was estimated by calculating the OR using the formula in the following:

(I) $$\text{OR=}\left(\text{a}/b\right)/\left(\text{c}/\text{d}\right)$$

Where a = Number of women with paternal lineage; b = Number of women with maternal lineage; c = Number of men with paternal lineage; and d = Number of men with maternal lineage.

This calculation reframes the question of the mode of transmission. Rather than asking “who is the parent” or “what is the parental lineage” (as in “maternal” vs. “paternal” transmission), it asks “which chromosomes might mediate the transmission of risk.” It seeks to isolate the risk imparted by the X chromosome. This method and its rationale, in terms of elucidating the genetic pathways of inheritance, are shown in Table 1.

The OR calculated using the method described in Table 1‘s legend may be used to estimate the RR conferred by the X chromosome, under several assumptions. First, this analysis method can be used if AD has a relatively low occurrence rate. In fact, the frequency of LOAD patients with a unilateral family history of disease was quite low, both in our clinic population^17^ and in the UPDS population.^6^ We consider that the OR calculation method described can control for several confounders, which may be expressed equally to influence disease occurrence in each of the four cells or each of the four cell pairs of the 2×2 table. These include non-nuclear-mediated modes of maternal effect on risk, such as mitochondrial transmission and gestational environment; any effect of the Y chromosome unrelated to the risk of developing AD or of the phenomenology or indirect effect of being a woman or a man; and most of the effects of imprinting mechanisms, as male and female probands are positioned equally to inherit a culpable autosomal chromosome from either father or mother and to have it expressed differently depending on whether it was inherited from father or mother. A recent review concludes that X chromosome inactivation (XCI) occurs randomly in humans, regardless of parental origin.^21^ Hence, the effects of XCI would impact women with LOAD equally, regardless of their family history. The Y chromosome-attributable risk for AD remains a moving target, and our assumption that we can ignore its role in the transmission of risk of developing AD in the present calculation may not withstand the test of time. A recent report indicates that mosaic loss of chromosome Y (mLOY), a common aging-related somatic event, confers an increased risk of developing AD.^22^ However, the genes used to calculate the polygenic risk score for mLOY are found to reside on autosomal chromosomes and could be transmitted equally from the father or mother. Completion of the sequencing of the Y chromosome,^23^ may broaden the opportunity to elucidate its role in normal brain development and the occurrence of neurodegeneration, including AD.

The estimated proportion of the total genetic risk carried by the X chromosome (expressed as a percentage) may be derived using the following formula:

Estimated proportion of genetic risk borne by the X chromosome =

(II) $$\text{Estimated}\;\text{proportion}\;\text{of}\;\text{genetic}\;\text{risk}\;\text{borne}\;\text{by}\;\text{the}\;\text{X}\;\text{chromosome}=\left(\text{OR}-1\right)/\text{OR}\times 100\%$$

The interpretation of the formula is as follows: an OR of 1.0 means that no risk resides on the X chromosome; an OR of 1.2 means that 17% of the risk resides on the X chromosome; an OR of 1.33 means that 25% of the risk resides on the X chromosome.

ORs, CIs, and *P*-values were calculated using an online calculator (https://www.medcalc.org/calc/odds_ratio.php).

The plan of analysis was developed (by C.A. and S.W.) before accessing the sex-specific UPDB data. We decided *a priori* that our primary analysis would be based on the 175 AD probands with SDRs affected by AD (father’s brother or sister vs. mother’s brother or sister) and that our secondary analysis would be based on the 1850 AD probands with TDRs affected by AD (father’s first cousins vs. mother’s first cousins). We did not calculate a combined OR or percent risk estimate because of the imbalance between the groups — the sample size used in the secondary analysis was more than 10 times larger than that in the primary analysis. We also did not perform an analysis based on the mother’s or father’s involvement because the total number of parents affected (40) was small (Table 3 of Cannon-Albright *et al*.^6^), largely due to the small window of view to AD deaths in the UPDB, from which only ICD-9 and ICD-10 coding was available.

### 2.3. Standard protocol approvals, registrations, and patient consent

The study was approved by the University of Utah Institutional Review Board and the Utah Resource for Genetic Epidemiological Research, which collectively oversees the use of UPDB data.^6^ Individual consent was not required to access the data.

## 3. Results

The data from Table 5 of the original publication^6^ are shown separately for female and male probands (with and without AD) (Tables 2 and 3).

### 3.1. Primary analysis

As shown in Tables 2 and 3, there were 175 AD probands (=25+11+42+26+21+8+25+17) with parental lineage of AD determined by SDRs; this proband group consisted of 104 women and 71 men. Their distribution by parental lineage is presented in Table 4.

### 3.2. Secondary analysis

As shown in Tables 2 and 3, there were 1850 AD probands (=582+629+296+343) with parental lineage of AD determined by third-degree relatives; this proband group consisted of 1211 women and 639 men. Their distribution by parental lineage is presented in Table 5.

### 3.3. Summary of results

These results do not support the role of genetic information carried on the X chromosome in conferring a risk for AD. The null result – the absence of support for a role for genetic information carried on the X chromosome in conferring a risk for AD – may have occurred because indeed no risk for AD is mediated by the X chromosome. We considered that the null result might be caused by an inherent error in our formula. The inherent assumptions in applying this formula are stated in the methods section. However, these assumptions do not account for the gap between the current findings and those of our previous study.^17^

## 4. Discussion

### 4.1. Preamble

Due to the paucity of loci predisposing to AD that had been identified on the X chromosome, we were prompted to develop a new method to estimate the risk for AD conveyed via the X chromosome,^17^ because of evidence that the risk for the disease may depend on the sex of the affected parent, and possibly on the sex of the offspring. The first application of this method indicated that the risk borne by the X chromosome might be considerable. We then decided to see if a similar result would be obtained in an existing dataset of patients with AD and a thorough history of the presence or absence of AD in their families, i.e., the UPDS. Our initial results did not replicate in the new dataset. In this section, we discuss several reasons why limitations in the second dataset may have biased the result toward “null,” and how the lessons learned may influence future research to advance understanding of the role of the X chromosome in conferring risk for AD.

### 4.2. Methodological critique

The magnitude of the difference between the results obtained in this and the original study^17^ suggests that a comparison of the populations between the two studies is warranted, specifically looking for factors that may bias the OR in the present study toward unity, that were not present in the original study. This comparison is presented in Table 6.

The chief difference between the two studies is that one had a population largely of carefully selected, newly diagnosed, incident patients and the other included a population of deceased individuals, in whom the cumulative lifetime risk to develop AD has been able to assert itself. Factors that impact the initial or early presentation (incidence) of LOAD, which are appropriate for consideration as risk factors, may not be detectable in a population of deceased individuals. The female-to-male ratio of the probands in the previous study (Cognitive Disorders Clinic) was closer to unity than that of the UPDB population, indicating that the Cognitive Disorders Clinic population tended to evaluate incident patients. We have previously^24^ pointed out that the female-to-male ratio of AD in population-based age-specific incidence studies was 1.0, and that it dropped to 0.6 when looking at population-based age-specific incidence of aMCI (usually an early clinical stage of what will evolve to be AD). The greater prevalence of AD in women is due to their longer lifespan, or the shorter lifespan of men who die at younger ages from competing causes of mortality. It is also likely that males documented in the UPDB, including those who also might have early manifestations of AD, died of other causes, and only these non-AD diagnoses were reflected in their death certificates.

The 20-year gap between the average age at presentation of the memory disorders clinic population and the age at death of the deceased population documented in the UPDB suggests that the clinic was seeing patients with relatively early-presenting LOAD, given that the usual average duration of the disease after diagnosis is 3 – 8 years. In the Baltimore Longitudinal Study of Aging, the median survival times ranged from 8.3 years for persons diagnosed as having AD at 65 years old to 3.4 years for persons diagnosed as having AD at 90 years old.^25^ Thus, the two studies reported on different populations: one on a population with relatively early onset LOAD, while another on a population with a much later onset of the disease. This idea is reinforced by the observation that the median age of the AD population in UPDB, which is 85 years (55% of whom died after 1978), is also higher than the average for the Utah population:^26^ In Utah, life expectancy at birth for males increased from 72.4 years in 1980 to 77.1 years in 2020, and for females from 78.6 to 80.9 years.^26^ This raises the possibility that some of the affected probands in the UPDB suffered from a recently-recognized limbic-predominant age-related TDP-43 encephalopathy (LATE),^27^ rather than AD. Limitations of using death certificate data to define cases of AD have been discussed previously.^6^

The third methodological difference is that while the affected probands identified from the clinic population did not include individuals with a bi-parental history of dementia, the available UPDB data did not permit the exclusion of such individuals. Thus, this would bias the OR in the UPDB data toward unity.

Another conspicuous difference has less to do with methods than with yield. Less than 1% of individuals identified with AD in the UPDB were identified as having a parent with AD, compared to over 50% of the cognitive disorders clinic population with a unilateral family history of AD or dementia. It is challenging to explain how overall low case finding of AD parents of probands with AD would affect the chances of finding male patients whose fathers had AD as compared to the chances of identifying male patients whose mothers had AD or female patients whose fathers or mothers had AD. However, the low number of patients in the clinic study was the main reason this population-based database study was conducted, and it is instructive to note that insofar as identifying large absolute numbers of parents of patients with AD who also had AD, we were not more successful with the population-based database. Furthermore, this is not a unique circumstance. We conclude that searching for risk factors for AD, including genetic risk factors, has severe limitations when performed in prevalent populations or deceased populations.

### 4.3. Implications for future research

#### 4.3.1. Future epidemiological studies

Pinpointing the role of an elusive risk factor, the X chromosome, will require that special efforts be invested in the methodology of the search. We suggest that a strategy based on looking for X-linked genetic risk in the subpopulation of LOAD patients with a unilateral family history of the disease may increase the yield of the search. Epidemiological evidence for the contribution of the X chromosome to the development of AD will need to be sought prospectively in incident patient populations with newly diagnosed aMCI or LOAD and a positive family history for an amnestic disorder. Contemporary studies should avail themselves of confirmation of the diagnosis of AD in the probands using biologic markers.^28^ A standardized protocol will be needed to acquire a family history from these patients. While family history may be limited in many patients, it may be positive in close to 30%; of these – perhaps upto three-quarters may report unilateral parental lineage, or 22% (absolute). A relatively small number of patients with unilateral parental lineage (several hundred) would be required to replicate, with greater sensitivity, the results of our earlier study.^17^

#### 4.3.2. Future genetic studies

A more ambitious goal of future research would be to seek genetic localization of X chromosome changes. Following the approach of relying on individuals with a unilateral family history of disease, a much larger number of incident cases are needed to compare the X chromosomes of men with a maternal family history of AD (that might carry some risks for the disease) to those of men with a paternal history of the disease (which would not be carrying risk factors for the disease). A modified design would be needed to perform a similar analysis in women with unilateral parental history of disease because 50% of their X chromosomes might not confer risk for disease. If each X chromosome could be identified as arising from the affected or the unaffected parent, it would be appropriate to compare the X chromosome from the affected female parent to that from the unaffected female parent. While the specifics of designing genetic studies are beyond the scope of this project, we suggest that looking for genetic risk factors in the subset of patients with a unilateral family history of LOAD may provide new insights into the sex-specific genetic causes of AD, which have been elusive thus far. A possible direction may be to focus on genes already known to be responsible for mental functions, which are represented disproportionately on the X chromosome, comprising perhaps 15% of the total genes responsible for mental functions,^29^ while only 4% of the total number of protein-coding genes in the genome are represented on the X chromosome.^30^ The X chromosome is represented disproportionately in the Online Mendelian Inheritance in Man (OMIM) listing of genes linked to mental retardation. It is estimated, using 2014 – 2016 data, that 22.3% of the total number of genes contributing to mental retardation reside on the X chromosome.^29,30^ The search to explain the role of the X chromosome in the development of AD may be advanced by focusing on genes residing on the X chromosome that already have been implicated in carrying a risk for neurological disease (listed in OMIM) by looking for minor changes in these genes in X chromosomes enriched for the likelihood of carrying risk by the unilateral parental family history of their bearers.

### 4.4. Clinical implications

Identifying the risk for AD carried by the X chromosome will permit refinement of the prognostic information regarding the lifetime risk of developing AD that can be given to the offspring of parents with AD, based on the sex of the affected parent and that of the offspring. Identifying the proportion of risk mediated by the X chromosome may serve as an impetus to develop the methodology to help identify the genetic loci mediating the risk.

If genetic risk factors can be identified as specific polymorphisms on the X chromosome, further refinement of prognostic information may be possible. In turn, once a complete map of genetic risk factors including those residing on the X chromosome is developed, it may be possible to stratify individuals based on their lifetime risk of developing AD and to use preventative and early therapeutic interventions in high-risk individuals, which are tailored to their genetic makeup. Besides, it may be possible to apply established genome editing techniques^31–33^ to modify polymorphisms to mitigate disease risk. A good understanding of the risk factors can also considerably benefit individuals carrying an increased genetic risk for AD. They may manipulate the modifiable individual^34^ and environmental^35^ risk factors for the disease so as to mitigate the risk for AD development.

## 5. Conclusion

At present, the precise mechanisms by which the risk of LOAD is transmitted genetically are incompletely clarified. Except for the ApoE4 allele, other polymorphisms convey very minimal risk. There are some indications that maternally-transmitted risk may be greater than paternally-transmitted risk, but the observation is not consistent, and the mechanism is unclear. There are additional indications that the transmission of risk to men may be different than the mode of transmission to women, although such findings are inconsistent from one study to another. If there is indeed a clear-cut difference in the mode of risk transmission, we can assume that the transmission is mediated by the X chromosome. At present, studies focusing on discovering risk-conferring polymorphisms on the X chromosome are outstripped by those on the 22 autosomal chromosomes. This is particularly puzzling because the X chromosome carries a disproportionate share of genetic material coding for neural network development, and is implicated in mental function and dysfunction.^29,30^ We, therefore, proposed a new method, developed based on an epidemiological approach and a unique approach to data analysis,^17^ to estimate the risk conferred by the X chromosome in a subpopulation of patients with LOAD who had a unilateral parental family history of dementia. When first applied to a population of newly diagnosed, incident patients, the analysis indicated that 70% of the risk was mediated by the X chromosome in this population. We then set out to replicate the findings in an existing database but were not successful. This led us to examine the differences between the populations of the original study^17^ and the present study. We saw that the inherent characteristics of this pre-existing dataset were greatly different from those of the incident dataset of the original study, in a way that biased the study toward producing a null result. Thus, it is necessary to focus future efforts on searching risk factors for LOAD on datasets of newly diagnosed incident cases, if there is to be a chance not only to replicate the findings of our original study but also to have a substrate for genetic analysis that truly carries in excess the risk factors for which we are looking. The implications are that the dataset would need to be assembled prospectively. A further implication of our approach is that the search for genetic risk factors on the X chromosome might become more fruitful if performed by comparing X chromosomes derived from a sample of individuals that is enriched for the likelihood for carrying pathogenic polymorphisms on chromosome X (men who had an affected maternal line) to X chromosomes from individuals with little risk of having the disease mediated by the X chromosome (men who had an affected paternal line). The corollary of these considerations, in our opinion, is that if these or similar approaches are not implemented, and we continue to look for genetic markers for LOAD on the X chromosome in the same way we have looked for them so far, we will continue to have limited success in finding them.

The introduction of new treatments for LOAD^36,37^ presents an exciting opportunity to enroll newly-diagnosed, biologically-confirmed incident cases into epidemiological studies of the disease’s inheritance patterns. Only a few hundred individuals with unilateral parental lineage would be needed to generate a robust estimate of the risk mediated by the X chromosome. A larger number of individuals with unilateral parental lineage for the disease would be able to contribute an enriched substrate necessary for identifying new pathogenic loci on the X chromosome.

The new directions proposed by the present research may lead to an improved understanding of the transmission of familial LOAD and the role played by the X chromosome.

## Figures and Tables

**Table 1.** Method of analysis based on ancestral family history, sex of the affected patients, and sex of the affected relatives.

Probands	Affected ancestral side	
	Paternal	Maternal
Women	a	b
	22A+X	22A+X
Men	c	d
	22A	22A+X

Notes: a: Number of female AD probands with father or/and paternal relatives affected by AD. Risk can be conferred by the 22 autosomal chromosomes and/or the X chromosome; b: Number of female AD probands with mother or/and maternal relatives affected by AD. Risk can be conferred by the 22 autosomal chromosomes and/or the X chromosome; c: Number of male AD probands with father or/and paternal relatives affected by AD. Risk can be conferred only by the 22 autosomal chromosomes. This feature is unique only to this group; d: Number of male AD probands with mother or/and maternal relatives affected by AD. Risk can be conferred by the 22 autosomal chromosomes and/or the X chromosome. The odds ratio (OR) is determined by finding the ratio of (a: b) to (c: d) and estimates the relative risk (RR) conferred by the X chromosome.

**Table 2.** Comparison of estimated RRs between paternal and maternal relationships, regardless of all other relationships, for female probands identified from UPDB

Family history	N	Obs	Exp	RR	*P*	95%	CI
≥1 maternal first cousin	18,884	582	523.37	1.11	0.01	1.02,	1.21
≥1 paternal first cousin	19,122	629	553.3	1.14	0.001	1.05,	1.23
**≥1 mother’s brother**	**745**	**25**	**17.04**	**1.44**	**0.07**	**0.93,**	**2.12**
**≥1 father’s brother**	**606**	**11**	**11.97**	**0.92**	**0.89**	**0.46,**	**1.64**
**≥1 mother’s sister**	**1339**	**42**	**31.9**	**1.32**	**0.077**	**0.95,**	**1.78**
**≥1 father’s sister**	**1163**	**26**	**25.0**	**1.04**	**0.84**	**0.68,**	**1.53**

Note: Data from second-degree relatives are presented in boldface.

Data from third-degree relatives are in regular font.

Abbreviations: N: Number of individuals; Obs: Observed;

Exp: Expected; RR: Relative risk; *P*: *P* value; 95% CI: 95% confidence interval (lower limit, upper limit).

**Table 3.** Comparison of estimated RRs between paternal and maternal relationships, regardless of all other relationships, for male probands identified from UPDB

Family history	N	Obs	Exp	RR	*P*	95%	CI
≥1 maternal first cousin	19,535	296	288.2	1.03	0.66	0.91,	1.15
≥1 paternal first cousin	19,922	343	305.2	1.12	0.03	1.01,	1.25
**≥1 mother’s brother**	**951**	**21**	**11.7**	**1.79**	**0.0098**	**1.11,**	**2.74**
**≥1 father’s brother**	**782**	**8**	**8.1**	**0.99**	**1**	**0.43,**	**1.95**
**≥1 mother’s sister**	**1650**	**25**	**20.1**	**1.24**	**0.31**	**0.80,**	**1.84**
**≥1 father’s sister**	**1450**	**17**	**15.5**	**1.09**	**0.80**	**0.64,**	**1.75**

Note: Data from second-degree relatives are presented in boldface.

Data from third-degree relatives are in regular font.

Abbreviations: N: Number of individuals; Obs: Observed;

Exp: Expected; RR: Relative risk; *P*: *P* value; 95% CI: 95% confidence interval (lower limit, upper limit).

**Table 4.** Analysis based on ancestral family history, based on the sex of the affected probands, and the sex of the affected parental side

Proband (n=175)	Lineage	
	Paternal (father’s brother or sister)	Maternal (mother’s brother or sister)
Female (104)	37 (11+26)	67 (25+42)
Male (71)	25 (8+17)	46 (21+25)

Notes: (1) The parental lineage of AD was determined by second-degree relatives (data taken from Tables 2 and 3). (2) The calculated OR is 1.01 (37:67/25:46), with a 95% CI of 0.54 – 1.91 (*P*=0.96).

**Table 5.** Analysis based on ancestral family history, based on the sex of the affected probands, and the sex of the affected parental side

Proband (n=1850)	Lineage	
	Paternal (father’s first cousins)	Maternal (mother’s first cousins)
Female (1211)	629	582
Male (639)	343	296

Notes: (1) The parental lineage of AD was determined by third-degree relatives (data taken from Tables 2 and 3). (2) The calculated OR is 0.93 (629:582/343:296), with a 95% CI of 0.77 – 1.13 (*P*=0.48).

**Table 6.** Comparison of populations and methods between the present study and the previous study^17^

Attribute	Present study (UPDB)	Previous study (Cognitive Disorders Clinic)^17^
Population	Deceased individuals in a database	Patients presenting to the clinic, mostly for initial diagnosis
Diagnosis	Based on death certificates	Based on clinical criteria to diagnose early disease
Percentage of probands in population from which they were drawn	3% (4436/149303)	22% (71/320)
Proband sex ratio (F: M)	64:36 = 2:1	40:31 = 1.3:1
Age-based inclusion criteria	No	Between 55 and 80 years at presentation
Proband age	85 years (median) at death (range 39 – 103)	66.1±5.1 years for women and 68.1±6.5 years for men at presentation (mean±standard deviation)
Unilateral parental family history	Methods do not permit the exclusion of “bilateral” cases	Methods specifically excluded 25 “bilateral” cases
Observation	Only 40 probands (<1%) had a parent with AD identified (14 fathers, 27 mothers)	In most probands, it was a parent who had AD/dementia

## Data Availability

Access to UPDB data are allowed with appropriate application and approval.
